# Ventilator-associated pneumonia: microbiological profile and mortality

**DOI:** 10.1186/cc10176

**Published:** 2011-06-22

**Authors:** G Castro, JAT Lisboa, JIV Carvalho, JN Bacelar, MM Ferreira, CLVM Miranda, ACP Carvalho, KRS Cruz, RM Salazar

**Affiliations:** 1 Centro Universitário do Maranhão (UNICEUMA), São Luis - MA, Brazil

## Background

Ventilator-associated pneumonia (VAP) is the second most frequent infection in American intensive care units (ICUs) and the most frequent in European ICUs, and its incidence and mortality rates are still high, despite the continuous advances in diagnosis and treatment techniques. Although its multiple etiologies and complex diagnosis breed divergence about its management approach.

## Objective

To evaluate the microbiological profile of patients with VAP admitted to the ICUs of two hospitals in São Luís - MA.

## Methods

A descriptive, analytic, retrospective study, with 1,072 patients admitted to ICUs of the hospitals Dr Carlos Macieira and Centro Médico Maranhense between January 2008 and December 2009. The patients were stratified by age, sex, infection type, identified pathogens and ICU stay outcome. Data were analyzed by the software Epi Info^® ^(version 3.5.1; 2008) and so was calculated the chi-square (X^2^) nonparametric test, with 5% significance level adopted.

## Results

It was verified that 31.6% of the patients had a polymicrobial infection and 68.4% acquired infection by monobacteria. Gram-negative bacilli showed up as the most common pathogens overall. The multidrug-resistant bacteria incidence was 51.3% and its correlation with VAP mortality and the means of days under mechanical ventilation of infected patients did not present statistical significance respectively.

## Conclusion

VAP has been pointed out as a manifold etiology disease, with high morbi-mortality indexes that do not change according to the etiologic agents.
